# The Effect of Carnosol, Carnosic Acid and Rosmarinic Acid on the Oxidative Stability of Fat-Filled Milk Powders throughout Accelerated Oxidation Storage

**DOI:** 10.3390/antiox10050762

**Published:** 2021-05-11

**Authors:** Katerina Tzima, Nigel P. Brunton, Noel A. McCarthy, Kieran N. Kilcawley, David T. Mannion, Dilip K. Rai

**Affiliations:** 1 Department of Food BioSciences, Teagasc Food Research Centre, Ashtown, D15 KN3K Dublin, Ireland; aikaterini.tzima@teagasc.ie (K.T.); dilip.rai@teagasc.ie (D.K.R.); 2 UCD Institute of Food and Health, University College Dublin, Belfield, D04 V1W8 Dublin, Ireland; 3 Department of Food Chemistry and Technology, Teagasc Food Research Centre, Moorepark, Fermoy, P61 C996 Cork, Ireland; noel.mccarthy@teagasc.ie; 4 Department of Food Quality and Sensory Science, Teagasc Food Research Centre, Moorepark, Fermoy, P61 C996 Cork, Ireland; kieran.kilcawley@teagasc.ie (K.N.K.); david.mannion@teagasc.ie (D.T.M.)

**Keywords:** fat-filled milk powders, carnosol, carnosic acid, rosmarinic acid, antioxidants

## Abstract

The in vitro antioxidant effects of the most potent antioxidants of rosemary, namely carnosol, carnosic acid and rosmarinic acid (c: ca: ra) were assessed in fat-filled milk powders (FFMPs) under accelerated conditions (40 °C and relative humidity (RH) 23%) over 90 days. Lipid oxidation was assessed in FFMPs by measuring peroxide values (PVs), thiobarbituric acid reactive substances (TBARS) and aroma volatiles using headspace (HS) solid-phase microextraction (SPME) coupled to gas-chromatography-mass spectrometry (GC-MS). The antioxidant potency of c: ca: ra exhibited a concentration-related effect (308 ppm > 200 ppm > 77 ppm), with the highest concentration being the most effective at controlling the formation of TBARS and PVs. At a concentration of 308 ppm c: ca: ra were particularly effective (*p* < 0.05) in inhibiting all the evaluated oxidation indices (primary and secondary) compared to the control samples, but in some cases less effectively (*p* < 0.05) than butylated hydroxyanisole: butylated hydroxytoluene (BHA: BHT) (200 ppm).

## 1. Introduction

Lipid oxidation can lead to flavour deterioration in many foods during their manufacturing and storage [1]. Traditionally, manufacturers have used antioxidants to retard the development of lipid oxidation. However, the use of synthetic antioxidants (i.e., BHA and BHT) for its inhibition has raised concerns about their safety as there is evidence supporting their potential toxicity and carcinogenicity [2]. Hence, industrial interest has been focused on the reduction or substitution of these synthetic antioxidants in order to align their products with consumer demand for “clean-label” foods [2]. The demand for new sources of antioxidants led to many investigations of medicinal plants [3]. Among them, rosemary is an herb that is currently commercially utilised as a source of natural antioxidant agents for food preservation purposes, and its efficacy has been ascribed to its high content in (poly)phenolic compounds [4]. Furthermore, previous studies have shown that its high antioxidant potential is mainly attributed to the presence of three antioxidant phenolics, namely carnosol, carnosic acid and rosmarinic acid [5]. 

Ireland is one of the major dairy export nations worldwide, with products such as FFMPs being considerably profitable contributors in the category of prepared exports [6]. As FFMPs can be subjected to harsh climatic conditions throughout their distribution [7], the maintenance of their storage stability is of paramount importance. Their manufacture encompasses the blending of skim milk powder with vegetable oil [8], and their oxidative stability is primarily a function of the composition of their unsaturated fatty acids (UFA). Vegetable oils contain UFA that are highly susceptible to free-radical induced reactions, leading to rapid oxidative reactions in the presence of oxygen (O_2_), even at low temperatures [9]. One of the commonly used oils in FFMPs is sunflower oil, and its stabilisation is considered challenging [10] due to its high concentrations of linoleic (C18:2) (63 g per 100 g) and oleic (C18:1) (20 g per 100 g) acids [2]. In a previous study, carnosic acid exhibited higher antioxidant action in sunflower oil than the synthetic antioxidants BHA and BHT [11]. Other authors have also examined the antioxidant potency of rosemary ethanolic extract (200 ppm) in sunflower oil under accelerated storage conditions (60 °C, 21 days), and found that it exhibited nearly an equivalent ability to retard lipid oxidation as compared to synthetic antioxidants (BHA, BHT and tert-butylhydroquinone (TBHQ)) [10]. Therefore, there is strong evidence to suggest that naturally sourced antioxidants could substitute synthetic antioxidants to effectively retard the onset of oxidative rancidity of FFMPs even under accelerate conditions. 

However, studies attesting to the antioxidant properties of whole plant extracts can be difficult to reproduce due to the natural variability regarding the antioxidants they contain and are often dependent on their source of location, stage in their growth cycle or different post-harvest storage times. Therefore, in the present study, a standardised additive ratio (0.25: 0.38: 0.37) of the most potent natural antioxidants normally found in rosemary, c: ca: ra, was utilised at three different total concentrations (77, 200 and 308 ppm) to assess its effectiveness in retarding oxidative deterioration of FFMPs with incorporated sunflower oil (26% *w/w*). Oxidative deterioration in FFMPs with added natural antioxidants was subsequently compared to that present in FFMP with the addition of synthetic antioxidants BHA: BHT (1:1) and control samples without the addition of antioxidants. FFMPs were stored under accelerated storage conditions (40 °C, RH 23%) for 90 days and assessed prior and after accelerated storage (0, 5, 13, 27, 41, 69 and 90 days). The protective effects of the different natural and synthetic antioxidants in stabilising FFMPs were tested by measuring their PVs, TBARS, and volatile profile with SPME-GC-MS analysis, whereas their particle size and colour (L*a*b) were also evaluated. To the best of authors’ knowledge, this is the first study that evaluates the antioxidant effect of c: ca: ra in FFMPs.

## 2. Materials and Methods

### 2.1. Samples and Reagents

Skim milk powder was collected from a local dairy company, and the same batch was used for the production of all FFMPs. Antioxidant-free refined sunflower oils were purchased from a local store (SuperValu, Dublin, Ireland), and stored in closed containers, in the dark under refrigeration (4 °C) until use. 

1,1,3,3-tetraethoxypropane (TEP), trichloroacetic acid (TCA), starch, potassium iodide, 37% hydrochloric acid (HCl), isooctane, BHA, BHT, rosmarinic acid, 1-pentanol, 2-heptanone, 2-pentanone, 3-octen-2-one, 2, 4-decadienal, hexanal, heptanal, octanal, pentanal, 2-nonenal (E), acetone, 2-nonanone, undecanal 2-methyl-4-pentanol, 2-methyl-3-heptanone, isovaleraldehyde and ethyl butyrate were purchased from Sigma-Aldrich (now Merck, Wicklow, Ireland), whereas carnosol and carnosic acid were obtained from Extrasynthese (Extrasynthese Co., Genay Cedex, France). Thiobarbituric acid (TBA) and sodium thiosulfate were obtained from VWR (VWR International Ltd., Dublin, Ireland). Isopropanol (PrOH) was acquired from Romil (Lennox Laboratory Supplies LTD, Dublin, Ireland) and *n*-hexane from Honeywell (Honeywell, Dublin, Ireland). Acetic acid was obtained from AppliChem GmbH (Darmstadt, Germany). Milli-Q^®^ water (H_2_O) (18 mΩ) (Merck Millipore, Molsheim, France) or deionized H_2_O was used throughout the experiments.

### 2.2. FFMPs Production

**Figure A1:**
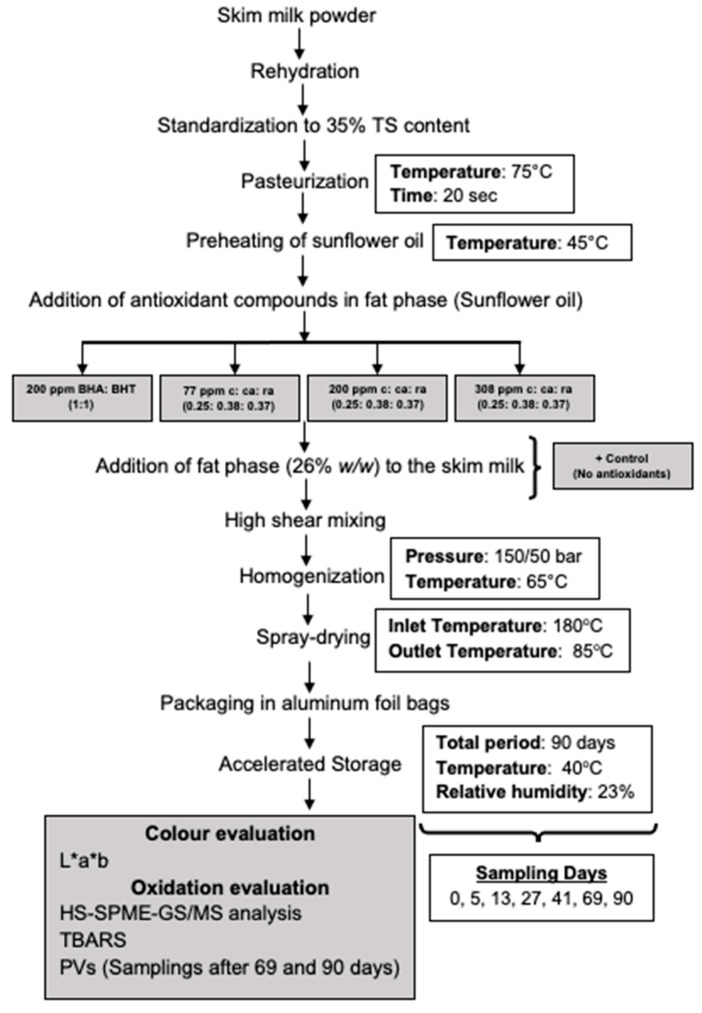
Schematic diagram of FFMP production with or without the addition of antioxidant compounds.

FFMPs were produced in the Moorepark Technology Limited pilot-plant facility (Fermoy, Co. Cork, Ireland). A flow chart illustrating their production and analysis is illustrated in Figure A1 (Appendix A). In summary, skim milk powder (~20 kg) was rehydrated to 35% total solids (TS) using a Y-Tron high shear mixer (YTRON Process Technology GmbH., Germany). The rehydrated milk was pasteurised at 75 °C for 20 s using a Microthermics^®^ UHT/HTSTL lab-25 EHVH (Raleigh, NC, USA) continuous-flow heater, before being cooled down to 45 °C. The pasteurised rehydrated skim milk was recombined with sunflower oil, with or without added antioxidants, to produce fat-filled milk of standardised fat content (26%, *w*/*w*). Control samples were produced without the addition of antioxidants, whereas the following combinations of synthetic and natural antioxidants were used, with their concentration to be based on the fat content of the FFMPs: (a) 200 ppm of 1: 1 BHA: BHT, (b) c: ca: ra (0.25: 0.38: 0.37) at the same additive concentration (200 ppm) as the mixture of BHA: BHT, (c) c: ca: ra (0.25: 0.38: 0.37) at an additive lower concentration (77 ppm) compared to the mixture of BHA: BHT, and (d) c: ca: ra (0.25: 0.38: 0.37) at an additive higher concentration (308 ppm) compared to the mixture of BHA: BHT. The ratio of c: ca: ra (0.25: 0.38: 0.37) was determined using the method of [12] for optimal recovery of the three compounds from freeze-dried rosemary. Prior to the addition, sunflower oil was heated in a heating cone to 45 °C, and the antioxidants were solubilised in it prior to their combination with the rehydrated skim milk. After combining the skim milk and sunflower oil using a high-shear mixer (Silverson, East Longmeadow, MA, USA), the mixture was homogenised using a 2-stage homogenizer (GEA Niro Soavi NS2002H, Twin Panda 400, Parma PR, Italy) at first- and second-stage pressures of 150 and 50 bar, respectively, at 65 °C. Each homogenised and supplemented with sunflower oil (with or without antioxidants) rehydrated skim milk was concentrated after spray drying with an Anhydro 750 Micraspray singe stage spray dryer (Anhydro, SPX Brand, WI, USA), with the air inlet and outlet temperatures set at 180 °C and 85 °C, respectively. FFMPs production was carried out in triplicate from 3 independent trials. The produced powders were then packed in unsealed aluminium foil sample bags under atmospheric conditions. The different FFMPs were then stored under accelerated conditions, at 40 °C, RH 23% and for 90 days, in a Memmert GmbH+Co.KG chamber incubator (Schwabach, Germany). Sampling was conducted prior to accelerated storage (day 0) and on days 5, 13, 27, 41, 69 and 90, after properly mixing each FFMP.

### 2.3. Particle Size Distribution Prior to Spray Drying

Particle size analysis of fat-filled milk concentrates (prior to drying) was performed in triplicate using static light scattering with a Malvern Mastersizer 3000 laser-light diffraction instrument (Hydro MV, Malvern Instruments Ltd., Worcestershire, UK) equipped with a 300 RF lens, following the protocol of Magan et al. [13]. Size distributions were determined by polydisperse analysis, and measurements were taken when the laser obscuration reached ~3%. In respect to the setting of the optical parameters, the particle and dispersant (H_2_O) refractive indices were equal to 1.46 and 1.33, respectively. The median (D_50_) and the cumulative diameters (D_90_ and D_10_) were measured to determine the span index through Equation (1), as given in the study of Buggy et al. [14]:

Span = (DV_0.9_ − DV_0.1_)/DV_0.5_ (1)

where: DV_0.9_ is the particle diameter below which 90% of the population of the distribution lies below, DV_0.1_ is the particle diameter below which 10% of the population of the distribution lies below and DV_0.5_ is the median particle diameter.

### 2.4. Colour Evaluation

L*, a* and b* represent lightness from black (0) to white (100), redness (+) or greenness (−), and yellowness (+) or blueness (−), respectively. The colour attributes (L*, a*, b*) of the FFMPs prior and throughout accelerated storage conditions were measured using a portable colorimeter (Konica Minolta, Ireland) after the samples had been vacuum-packed. The colour of each sample was measured randomly 3 times on the surface of each bag.

### 2.5. PVs

Fat was extracted according to Kim, Chen and Pearce [15] in triplicate for the estimation of PVs. A total of 4 g of each FFMP after 69 and 90 days of accelerated storage (highly oxidized samples) was weighed and reconstituted with 16 mL of Milli-Q H_2_O at 40 °C, with vortexing for 2 min. Subsequently, 70 mL of n-hexane: PrOH (3:1 *v/v*) was added to the powder and the mixture was stirred for 15 min, centrifuged (1000 g) and decanted. The organic phase was collected and 30 mL of n-hexane: PrOH (3:1 *v/v*) was added in the aqueous phase. The collected organic phase was evaporated at room temperature under nitrogen. 

Subsequently, PVs were determined based on the ISO3960 method [16] for vegetable oils in triplicate. The previously extracted fat from FFMPs was weighed and solubilised in a mixture of 3:2 acetic acid: isooctane (3 mL: 0.5 g fat matter). Subsequently, a saturated solution of potassium iodide (50 μL: 0.5 g) was added. The vessel was closed and after mixing, it was stored in the dark for 5 min. Milli-Q H_2_O (3 mL: 0.5 g) and 1% *w*/*v* of freshly prepared starch (50 μL: 0.5 g) was added, the mixture was titrated with 0.01 N sodium thiosulfate. Results were expressed as milliequivalents (meq) per kg of fat matter according to Equation (2):

PV = (V_1_ − V_0_) × C × 1000 × T/m (2)

where: V_1_ is the consumed sodium thiosulfate solution in the main test (mL), V_0_ is the consumed sodium thiosulfate solution in the blank test (mL), C is the molar concentration (molarity) of the sodium thiosulfate solution (mol/L), T is the titer of the thiosulfate solution and m is the weighed oil quantity (g). 

Reagent quality was confirmed after a blank titration under the same conditions, where no more than 0.5 mL of sodium thiosulfate solution was consumed. 

### 2.6. TBARS

Secondary oxidation was measured using the TBARS method according to the protocol of Buege and Aust [17] with the modifications of Burri et al. [18]. Briefly, 0.5 mL of reconstituted FFMP in Milli-Q^®^ H_2_O (0.125 g in 0.5 mL) was mixed with 2.5 mL of a solution containing 0.75% TBA (*w/v*), 15% TCA (*w/v*) and 0.25 M HCl in triplicate. The mixture was then placed in a water bath at 90 °C for 10 min to develop a pink colour. Subsequently, the samples were cooled in H_2_O to stop the reaction process and were centrifuged at 3600× *g* for 20 min (Sorvall Lynx 6000, ThermoFisher Scientific, Waltham, MA, USA). The absorbance of the supernatant was measured at 532 nm using a spectrophotometer (Shimadzu UV-1700, Shimadzu Corporation, Kyoto, Japan). Absorbance measurements at 600 nm were also used as a correction factor for nonspecific turbidity in the samples. A standard curve was prepared using TEP. Results were expressed as μM MDA/g of FFMP according to Equation (3):

TBARS (μM MDA/g of FFMP) = [(Abs_534_ −(Abs_600_ + B)) − m]/(k × w) (3)

where: m and k values were obtained from the standard curve equation given by: y = kx + m, after plotting the difference in concentration TEP standard solutions versus the measured absorbances at 534 nm; Abs_534_ is the absorbance at 534 nm, Abs_600_ is the absorbance at 600 nm, B is the blank absorbance, and w is the sample weight (g).

In the protocol of Buege and Aust [17] 0.375% TBA is used. However, Burri et al. [18] showed that by using this TBA concentration, orange chromogens were formed, exhibiting a strong absorbance at 453 nm and a weak one at 534 nm, where MDA was measured. Hence, after assessing different concentrations of TBA, the authors concluded that by utilising twice the concentration of the reagent, a more intense pink colour was formed, and the orange chromogens were decreased. Therefore, these were the TBA concentrations that used in this study. 

### 2.7. HS-SPME-GC-MS Analysis

HS-SPME-GC-MS was conducted according to the method described by Clarke et al. [19], where SPME-based collection of volatiles was performed using a 2 cm, 50/30 μm, CarboxenTM/divinylbenzene/polydimethylsiloxane Stableflex fibre (Agilent Technologies Ltd., Castleview, Cork, Ireland). 1.25 g of sample was added to a 20 mL screw-capped SPME vial together with 0.25 mL of internal standard (ISTD) solution and 3.5 mL of distilled H_2_O. The ISTD solution contained 2-methyl-4-pentanol, 2-methyl-3-heptanone, isovaleraldehyde and ethyl butyrate at a concentration of 1 ppm in distilled H_2_O for samples before accelerated storage till the day 41, and 10 ppm concentration for those after 69 and 90 days of storage at accelerated oxidation conditions to account for the higher concentration of lipid derived volatiles on those sampling days. The vial was equilibrated to 40 °C for 10 min with pulsed agitation for 5 s at 500 rpm. Sample introduction was accomplished using a Bruker CombiPal autosampler (Elementec Ltd., Maynooth, KE, Ireland). The samples were analysed in triplicate. 

For calibration experiments, standard addition was performed, where 0.25 mL of a standard (STD) mix containing the 13 target compounds (1-pentanol, 2-heptanone, 2-pentanone, 3-octen-2-one, 2,4-decadienal, hexanal, heptanal, octanal, pentanal, 2-nonenal (E), acetone, 2-nonanone and undecanal) was added to 1.25 g of the sample together with 0.25 mL ISTD and 3.25 mL of distilled H_2_O. The STD mix and the ISTD were added at concentrations of 200–1600 µg/L and 1 mg/L in distilled H_2_O, respectively. These compounds were selected as they comprise some of the main compounds arising from the oxidation of sunflower oil (hexanal, 2,4-decadienal, pentanal, 1-pentanol, 2-heptanone, 2-pentanone, octanal, pentanal) [20] and milk powders (including those previously mentioned for sunflower oil in addition to 3-octen-2-one, 2-nonenal (E), acetone, 2-nonanone and undecanal) [19,21]. For samples after 69 and 90 days of storage, the concentrations were increased to 1–20 mg/L for the STD mix and to 10 mg/L for the ISTD. Five-point calibration curves were established for each target compound based on their relative responses against each associated ISTD. 

The SPME fibre was exposed to the HS above the samples for 45 min at a depth of 1 cm at 43 °C, automated by a Bruker CombiPal autosampler (Elementec Ltd., Ireland). Subsequently, the fibre was retracted and injected into the GC inlet and desorbed for 3 min at 250 °C. Injections were made on a Scion 456-GC (Elementec Ltd., Maynooth, Co. Kildare, Ireland) with an Agilent mid-polar DB-624 UI column (60 m × 0.32 mm × 1.80 μm) (Agilent Technologies Ireland Ltd., Little Island, Co. Cork, Ireland) using a split/splitless injector fitted with a merlin microseal and operated in split mode (ratio 10: 1). The column oven temperature was set at 65 °C, where it was held for 6 min, and, subsequently, it was increased at 10 °C/min to 260 °C and held for 4 min, yielding a total GC run time of 29.5 min. The carrier gas was helium and was held at a constant flow (1.0 mL/min). The detector was a Bruker EVOQ TQ MS (Elementec Ltd., Maynooth, Kildare, Ireland) running in single quad mode. The ion source and interface temperature were set at 260 °C. The MS mode was electronic ionisation (70 V) with the mass range scanned between 35 and 250 amu. Compounds were identified using mass spectra comparisons to the NIST 2014 MS library, and an in-house library constructed using the target compound STD mix. An auto-tune of the GC-MS was carried out prior to the analysis to ensure optimal GC-MS performance. 

### 2.8. Statistical Analysis 

In each case, 3 independent FFMPs were evaluated. The statistical analysis was performed on the mean values of the assessed technical replicates after the independent analysis of each FFMP, and the results were reported as mean ± SD (*n* = 3). Normality and homogeneity of variances were assessed with Shapiro–Wilk and Leven’s tests, respectively. Kruskal–Wallis followed by Stepwise-stepdown comparisons were employed when data did not follow a normal distribution (*p* < 0.05), while Welch’s analysis followed by Games–Howell test were employed in case of non-homogeneity of variance (*p* < 0.05). Spearman-Rho correlation (*r_s_*) coefficients were used due to lack of normal distribution (*p* < 0.05) in the mean values of all the FFMPs (*n* = 3) to reveal any relationship between the results of the TBARS and the quantified volatile compounds after HS-SPME-GC-MS. The significance level of all tests was set as 0.05 unless it was specified otherwise. Statistical analysis was carried out using SPSS Statistics, Version 24 (IBM Corp., Armonk, NY, USA).

## 3. Results and Discussion

### 3.1. Particle Size Distribution Prior to Spray Drying 

The particle size distribution of a milk powder not only affects its appearance, but also its reconstitution, flow properties and its surface reactivity [22]. As particle size can be influenced by the original milk characteristics [22], it was important to evaluate whether there was any difference (*p <* 0.05) in the particle size distribution of the FFMPs after adding different concentrations and types of antioxidants. Therefore, laser diffraction was used to characterise the emulsion particle sizes after oil addition, with or without antioxidants, and prior to spray drying. The amplitude of size distribution is commonly estimated after determining the span index of a matrix [22]. As demonstrated by the span index of the different FFMPs, in Table 1, the size distribution of particles in the different samples prior to spray drying was not significantly (*p* > 0.05) different. Hence, the evaluated samples were homogenous, and the addition of antioxidant compounds did not influence fat droplet size (*p* > 0.05).

### 3.2. L*a*b Colour Determination of FFMPs before and after Accelerated Storage

Colour is an essential indicator of food quality and is critically important for consumer acceptance. The colour FFMPs (L*a*b) after accelerated storage were not highly different from that of samples tested after production and prior to storage (Table 2). The maintenance of colour stability during storage could be due to the relatively low RH (23%) used during this study. Initiation of browning (Maillard) reactions takes place when water activity (aw) was above 0.2 (20% RH), whereas at 0.6 aw (RH 60%), the reaction rates reached their peak [23]. Therefore, the rate of this type of reaction was considerably low at the RH used in this study (23%). At the same time, oxidation reactions were reduced as aw was decreased in a food product. However, when aw was below 0.4 (RH 40%), the reaction rates begin to increase again, and, therefore, at an RH of 23%, it can be substantially high [23]. As it is illustrated in Table 2, the L* values of the different FFMPs prior to accelerated storage were not significantly (*p* > 0.05) different. The same trend (*p* > 0.05) was also observed for their a* and b* values on the same sampling day. Indeed, L* values were generally stable throughout accelerated oxidation. However, statistically significant differences (*p* < 0.05) were observed between the samples after 41 and 90 days of accelerated oxidation storage. After 41 days, a significantly lower (*p* < 0.05) lightness (96.7) was observed for the samples supplemented with 308 ppm of c: ca: ra in comparison to the samples with 200 ppm of BHA: BHT (97.5). However, none of these samples was significantly (*p* > 0.05) different from the control and the FFMPs with lower levels of phenolic compounds. After 90 days of storage, FFMPs with 200 and 308 ppm of BHA: BHT had significantly (*p* < 0.05) lower L* values compared to BHA: BHT (97.4), which were equal to 96.5 and 96.6, respectively.

Furthermore, there was a statistically significant (*p* < 0.05) effect of the different concentrations of c: ca: ra on the green to red colour coordinate a* values (5, 13, 27, 41 and 69 days of accelerated storage), as well as blue-to-yellow coordinate b* values (5, 13, 27, 41 and 90 days of accelerated storage) between the different FFMPs as shown in Table 2. For example, whilst prior storage, both a* and b* values were not statistically (*p* > 0.05) different, in all the evaluated FFMPs 69 days of storage, a* values of FFMPs with 308 ppm of c: ca: ra had a significantly (*p* < 0.05) higher redness (−2.8) compared to those with 200 ppm of BHA: BHT (−2.7) as well as the control samples (−2.5). However, with respect to the FFMPs tested after 90 days of accelerated storage, b* values also exhibited significant effects among samples as a significantly higher yellow colour was observed in control samples (10.2) and those with 77 ppm of c: ca: ra (10.4) in comparison to the samples with 200 ppm of BHA: BHT (9.3). The latter, however, did not differ significantly (*p* > 0.05) from those with 200 ppm (9.7) and 308 ppm (9.7) of c: ca: ra. In a previous study by Nielsen, Stapclteldt, and Skibsted [24], whole milk powders stored under accelerated conditions at 50 °C and RH 31% for 49 days also turned progressively darker and more yellow pigmentation, with increasing storage time [24]. The authors inferred that even if the rate of Maillard reactions, which form brown pigments throughout heat treatment, storage and exposure of milk powders to high temperatures [21] were reduced with the use of a low RH, they may still have slightly contributed to the colour differences of the different FFMPs after accelerated storage, and particularly after 90 days. 

### 3.3. PVs of FFMPs before and after Accelerated Storage 

**Figure 1:**
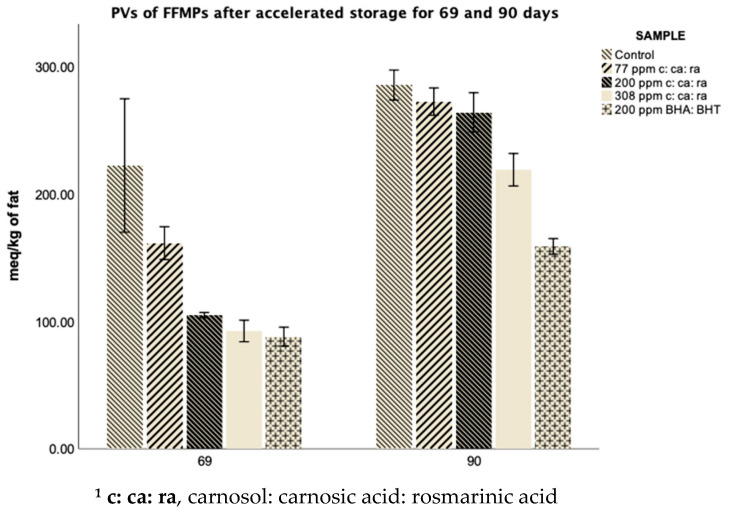
PVs of the FFMPs after accelerated storage (40 °C, RH 23%) for 69 and 90 days, with or without the addition of antioxidants. Values are the mean of three replicates (*n* = 3) ± SD ^1^.

The measurement of PVs constitutes a common method for the determination of primary oxidation products such as peroxides (LOO) and hydroperoxides (LOOH) in the initial stages of lipid oxidation for assessing the oxidative rancidity of fats and oils [25]. Cesa et al. [26] showed that the use of 3: 1 of *n*-hexane: PrOH, followed by iodometric titration yield the most representative results in the estimation of PVs in infant formulas when compared to other fat extraction protocols [26]. Hence, this protocol was also employed in our study, and the subsequent iodometric titration finally yielded PVs in the same range as those previously reported [11]. The influence of antioxidants during accelerated storage on PVs of FFMPs is shown in Figure 1 and Table A1 (Appendix A). PVs were determined only for the samples after 69 and 90 days of accelerated storage, as both TBARS values and volatile oxidation compounds were negligible before, as shown in the subsequent sections. Therefore, as only these two sample sets exhibited substantial oxidation rates, they were the only powders considered satisfactory for highlighting the clear relationships between the different types of FFMPs. 

Results indicated that PVs increased with storage time, exhibiting higher values for all the samples after 90 days of accelerated storage in comparison to 69 days. In addition, control samples had the highest PVs, after 69 and 90 days of accelerated storage. In the study of Zhang et al. [11], where the oxidation rate of sunflower oil samples was assessed using PVs in the presence or absence of antioxidants at 60 °C for 21 days, control samples reached a maximum PV value equal to 272 meq/kg. Even though different conditions and methodology were applied in the present study, our control PVs were in the same range after 90 days of accelerated storage at 40 °C, with a PV equal to 285.4 meq/kg. On day 69, the PVs of control samples were 222.22 meq/kg, followed by the rest of the samples in the following descending order: 77 ppm of c: ca: ra (161.33 meq/kg) > 200 ppm of c: ca: ra (104.89 meq/kg) > 308 ppm of c: ca: ra (92.44 meq/kg) > 200 ppm BHA: BHT (88.00 meq/kg). Statistical analysis revealed that control samples did not differ significantly (*p* > 0.05) from those containing 77 and 200 ppm of c: ca: ra. The lack of significance could be attributable to the fact that one of the three evaluated replicates in the case of control samples was highly skewing the data. However, this result is not surprising while assessing real-food samples and not controlled model systems. Particularly, in the case of control samples, where no antioxidants had been previously added, the oxidation rate on the surface of the FFMP can be substantially higher than that of the inner parts during storage. Hence, even if proper mixing was conducted prior to sampling, a higher level of oxidation was measured for this replicate, leading to this effect. However, a higher concentration of these phenolic compounds (308 ppm) seemed to effectively retard lipid deterioration of FFMPs as they exhibited a non-significant (*p* > 0.05) difference from those containing 200 ppm of BHA and BHT. 

After 90 days of accelerated storage, samples containing 200 ppm of BHA: BHT gave rise to the lowest values (158.8 meq/kg) compared to all the evaluated FFMPs, similarly to the samples received after 69 days. However, in that case, PV levels were significantly lower (*p* < 0.05) compared to 308 ppm of c: ca: ra, which exhibited the second-lowest PVs after 90 days of accelerated storage (219.9 meq/kg). Nonetheless, 308 ppm of c: ca: ra resulted in significantly (*p* < 0.05) lower PVs than control samples. On the other hand, 77 ppm (272.4 meq/kg) and 200 ppm of c: ca: ra (264 meq/kg) did not result in a significant (*p* > 0.05) difference in PVs in comparison to the control samples. 

PVs followed similar trends to the results of HS-SPME-GC-MS analysis and TBARS assessment as presented in Section 3.4 and Section 3.5. However, as is apparent from examination of Figure 1, even if the ranking of the different samples was similar, differences in PVs were not as apparent after 90 days of storage as those estimated after 69 days. This could be potentially attributed to the fact that most antioxidants remain effective during a certain period of time, whereas after that period, lower effectiveness was observed till the point that they entirely lose their efficacy [27]. As it could be concluded, the effectiveness of the employed phenolic compounds (c: ca: ra) to inhibit lipid peroxidation, exhibited a concentration-related effect, with the highest concentration (308 ppm) resulting in the lowest PVs compared to the second-highest (200 ppm) and finally the lowest (77 ppm). A similar effect was observed in the previously stated study of Zheng et al. [11], where the higher the concentration of carnosol was, the better the antioxidant potential in the sunflower oil samples [11]. However, even if we received reproducible results, the classic iodometric method was not without weaknesses. Due to the fact that PVs determination is highly empirical, variations in the procedure may generally affect the results [16].

### 3.4. TBARS of FFMPs before and after Accelerated Storage 

**Figure 2:**
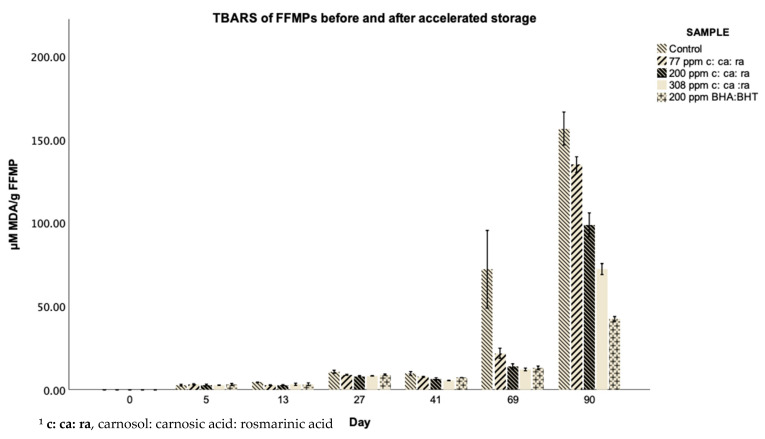
TBARS of the FFMPs before and after accelerated storage (40 °C, RH 23%), with or without the addition of antioxidants. Values are the mean of three replicates (*n* = 3) ± SD ^1^.

TBARS were defined as the quantity of malondialdehyde (MDA) (in mg) present in 1 kg of sample and were used as indicators of secondary lipid oxidation [11]. As illustrated in Figure 2 and Table A2 (Appendix A), TBARS values for samples with added antioxidants were clearly different after 69 and 90 days of accelerated storage and indeed were substantially lower after 41 days as compared to untreated control samples. However, even if a number of significant differences were observed after 13 and 27 days in the different FFMPs, clear trends that enabled their detailed comparison were only observed in the highly oxidized samples after 69 and 90 days of accelerated storage. TBARS of the FFMPs were strongly correlated (*r*_s_ ≥ 0.650, *p* < 0.01) with levels of the individual volatile compound, as shown in Table A3 (Appendix A). 

Both TBARs and oxidatively derived volatiles have been routinely used to assess secondary oxidation in lipids, and, therefore, this correlation was not surprising and has been reported by other authors [28]. After 69 days of accelerated storage, control FFMPs gave rise to the highest TBARS values equal to 72.14 μM MDA/g FFMP, followed by those with 77 ppm of c: ca: ra (21.76 μM MDA/g FFMP). FFMPs with 200 ppm of added c: ca: r resulted in the third-highest content of MDA, exhibiting a value of 13.95 μM MDA/g FFMP. Finally, the addition of 308 ppm c: ca: ra led to a lowest TBARS value that was equal to 12.16 μM MDA/g FFMP; however, not significantly (*p* > 0.05) lower in comparison to that of 200 ppm of BHA: BHT (13.07 μM MDA/g FFMP). However, again as a result of the high degree of variability, only FFMPs supplemented either with 308 ppm c: ca: ra or 200 ppm BHA: BHT had significantly (*p* < 0.05) lower values than the control FFMPs. After 90 days of accelerated storage, an apparent effect was observed, indicating that 200 ppm of BHA: BHT led to the lowest (42.42 μM MDA/g FFMP) oxidative deterioration of the samples. Samples with the highest concentration of phenolic compounds (308 ppm) had the second-lowest oxidation rate (72.27 μM MDA/g FFMP), followed by those with the same concentration as BHA: BHT (200 ppm) with values equal to 98.81 μM MDA/g FFMP. With respect to the samples with the lowest concentration (77 ppm) of phenolic compounds, higher TBARS values of 134.94 μM MDA/g of FFMP were observed. Control samples exhibited significantly higher (*p* < 0.05) oxidation (156.55 μM MDA/g FFMP) compared to all the samples that were supplemented with natural or synthetic antioxidants, whereas their MDA concentration was more than two times higher in comparison to the control samples tested after 69 days of accelerated storage. However, except for reducing the shelf-life and nutritional value of food formulations, secondary oxidation products are toxic at high levels [29]. 

The effect of rosemary and its major compounds on reducing the formation of MDA has been previously documented. Zhang et al. [11] stated that carnosic acid was capable of reducing the formation of MDA in sunflower oil when used at a range of concentrations. In the same study, when the oxidation rate of sunflower oil samples was assessed with TBARS during the presence or absence of antioxidants at 60 °C for 21 days, control samples reached a maximum TBARS value equal to 216 μΜ MDA/g [11]. Despite the fact the aforementioned authors’ employed different conditions and methodology than those applied in our study, our control TBARS were in the same range after 90 days of accelerated storage at 40 °C, with a previously stated TBARS value (156.55 μM MDA/g). 

All the samples were also significantly (*p* < 0.05) different to each other after 90 days of accelerated storage. However, even if 308 ppm of c: ca: ra yielded significantly (*p* < 0.05) higher TBARS values than the samples containing 200 ppm of BHA and BHT, this concentration still resulted in a greater than 50% reduction in MDA compared to the control samples. ring with 

Redondo-Cuevas et al. [30] also concluded that potent antioxidant components present in rosemary, including carnosol, carnosic acid and rosmarinic acid were probably responsible for lower MDA formation in rapeseed oil stored after addition of varying concentrations of rosemary powder (0.25–2% *w/w*) under accelerated conditions (120 °C for 1.5 and 3 h) as compared to the control samples. According to Senanayake [31], synthetic phenolic based antioxidants, such as BHA and BHT, which are comprised of one aromatic one hydroxyl (OH) group, only have the capacity to donate one hydrogen (H_2_). However, two OH groups were present in the aromatic rings (C11 and C12 positions) of both carnosol and carnosic acid. Hence, due to their structural superiority, these two compounds should have an intrinsically greater ability to scavenge free radicals and thus, retard lipid oxidation than BHA and BHT [31].

### 3.5. Volatile Secondary Oxidation Products in FFMP’s

Odour is an essential index for assessing the quality of edible oils and products containing them [32]. The primary UFA oxidation products are LOOH [33], which arise from a reaction between an unsaturated lipid and O_2_ and rapidly decompose into volatile and non-volatile constituents, including aldehydes (i.e., hexanal, hexenal, pentanal), ketones and hydrocarbons (i.e., pentane) that can negatively influence the aroma of a product [33]. In addition, the presence of aldehydes can also result in toxicological effects [29]. SPME is a rapid, sensitive, solventless, and cost-efficient method for the preparation of samples for GC analysis and is commonly applied for the determination of volatile compounds in fresh and oxidized oils [32]. In this study, HS-SPME-GC-MS analysis was conducted to determine the levels of 13 volatile compounds, known to arise from the oxidation of UFA, namely 1-pentanol, 2-heptanone, 2-pentanone, 3-octen-2-one, 2,4-decadienal, hexanal, heptanal, octanal, pentanal, 2-nonenal (E), acetone, 2-nonanone and undecanal, before and after accelerated storage of the FFMPs. 

**Figure 3:**
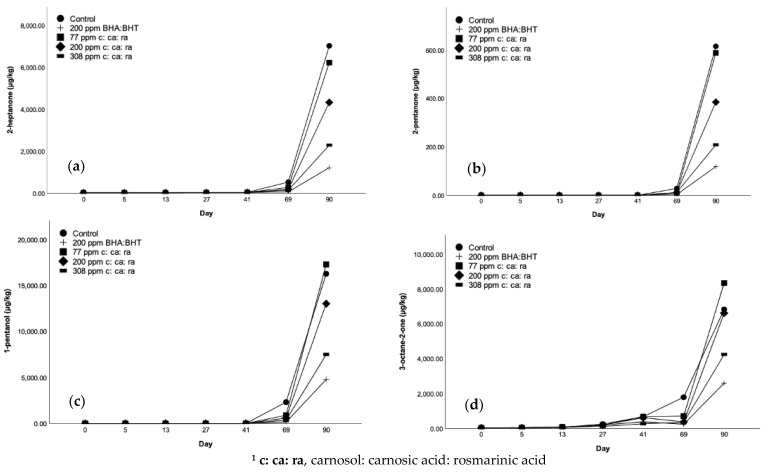

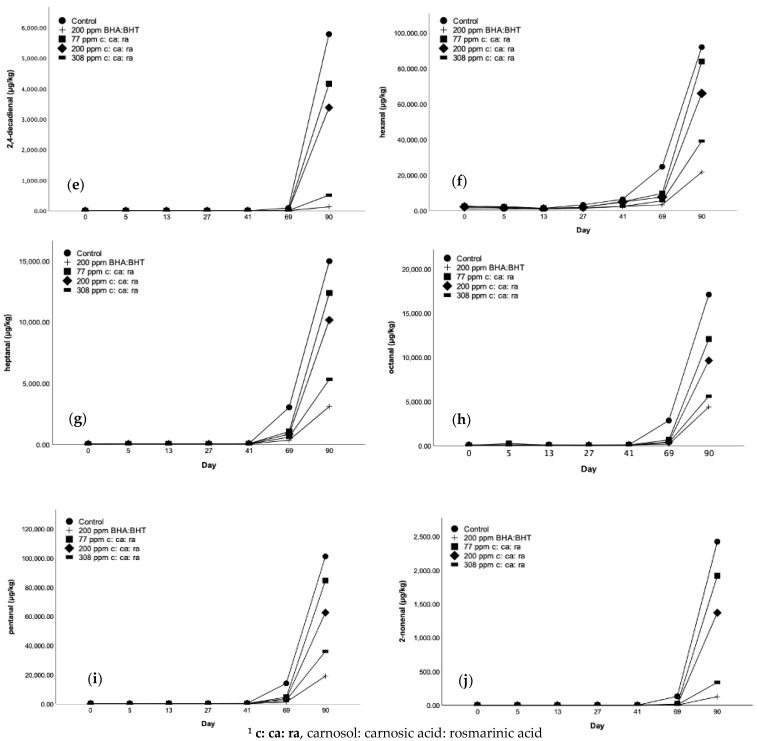
Levels of volatile compounds including (**a**) 2-heptanone, (**b**) 2-pentanone, (**c**) 1-pentanol, (**d**) 3-octane-2-one, (**e**) 2, 4-decadienal, (**f**) hexanal, (**g**) heptanal, (**h**) octanal, (**i**) pentanal and (**j**) 2-nonenal after HS-SPME-GC-MS analysis of FFMPs, before and after accelerated storage (40 °C, RH 23%), with or without the addition of antioxidants. Values are the mean of three replicates (*n* = 3) ^1^.

The formation of the key volatile oxidation compounds in FFMPs was affected by the absence or presence of different antioxidants. As it was initially revealed after assessing the volatile composition among the different samples, acetone, 2-nonanone and undecanal were absent in all cases, irrespective of the sampling day. HS-SPME-GC-MS revealed clear differences between the results obtained before and after accelerated storage, as illustrated in Figure 3a–j and Table A4, Table A5, Table A6, Table A7, Table A8, Table A9 and Table A10. For all treatments, there was, as expected, a gradual increase in lipid oxidation derived in volatiles as the samples progressed through accelerated storage. The highest concentration compound measured in most batches was hexanal, which ranged from 730 to 92,143 μg/kg, and was approximately 2 to 10 times higher than any other quantified compound. This was expected given the fact that C18:2 is the most abundant UFA in sunflower oil, hexanal is the main volatile secondary oxidation product resulting from its oxidation [2]. This effect was observed after all the sampling times (0 to 69 days), except after 90 days of accelerated storage, after which the samples had higher levels of pentanal, ranging from 19,117 to 101,202 μg/kg as opposed to a range of 21,672 to 92,143 μg/kg for hexanal. 

Levels of pentanal, hexanal and heptanal are good indicators of lipid oxidation in FFMPs [21], whereas hexanal has been reported as the major compound arising from lipid oxidation of milk powders [34]. As it was previously stated, samples prior to and during accelerated storage till sampling day 41 had considerably lower concentrations of all the different volatile compounds when compared to the samples tested after 69 and 90 days of accelerated storage (40 °C, RH 23%). Furthermore, 4 out of 10 quantified compounds, namely 1-pentanol, 2-pentanone, 2,4-decadienal and 2-nonenal (E), were absent prior to 69 days of storage. Therefore, the main effects of the absence or presence of the different antioxidant compounds was most striking in the last two sampling days. As illustrated in Figure 3i and Table A9 (Appendix A), pentanal content was 14,164 μg/kg for the control samples, followed by the samples containing 77 ppm c: ca: ra (4713 μg/kg), those with 200 ppm c: ca: ra (3616 μg/kg) and 308 ppm c: ca: ra (2646 μg/kg) and finally those with 200 ppm of BHA: BHT (1331 μg/kg). In all cases, levels of pentanal and hexanal were significantly (*p* < 0.05) reduced in powders supplemented with antioxidants in comparison to the control samples. Indeed, after 90 days of accelerated storage (Figure 3i), Table A10 of Appendix A), the highest pentanal concentration was again observed in the control samples and was equal to 101,202 μg/kg, equating to approximately 10 times higher levels compared to the control samples after 69 days of accelerated storage. Similarly, the lowest concentration was found in the samples containing 200 ppm of BHA: BHT (19,117 μg/kg). The second-lowest concentration (36,138 μg/kg) of pentanal was present in the samples containing the highest concentration of phenolic compounds (308 ppm), followed by those containing the same concentration (200 ppm) as BHA: BHT (62,673 μg/kg), and finally the samples containing a lower (77 ppm) concentration (84,638 μg/kg) of phenolics in comparison to BHA: BHT. However, the high variability of pentanal in the control samples after 90 days of accelerated storage led to a lack of statistically significant differences (*p* > 0.05) after comparison with the supplemented with antioxidants samples. However, a clear trend was observed with respect to their efficiency and the potential of all the different antioxidant combinations in reducing pentanal levels. Particularly, 308 ppm of c: ca: ra could considerably reduce its concentration, even if this reduction was significantly (*p* < 0.05) lower than that of 200 ppm BHA: BHT, which exerted a superior effect compared to all the different phenolic concentrations. 

Hexanal is recognized as one of the main lipid oxidation indicators in many samples as it is usually present in high concentrations throughout storage [28,33]. Hexanal is formed via the oxidation of C18:2 by the 13-LOOH [33]. According to Beliz et al. [35] the autoxidation of 2,4-decadienal also leads to the formation of hexanal and additional volatile compounds. Pentanal, which also arises from the oxidation of C18:2 in this case from the decomposition of 14-LOOH was also present in substantial quantities in all the treatments [35]. Oxidatively derived volatile compounds have been ascribed a range of odour descriptors and their potency is dependent on their odour threshold. For example, hexanal has been ascribed as an off-flavor of “grassy” [33] and its odour thresholds vary significantly in different matrices [35]. The odour threshold for hexanal in H_2_O is equal to 4.5 μg/kg [33], whereas a higher value of 12 μg/kg has been reported by Beliz et al. [35]. On the other hand, the odour threshold of pentanal in H_2_O has been reported to be 18 μg/kg [35]. However, the odour threshold of various volatile compounds is greater in oil and fat in comparison to H_2_O and air, probably as due to the matrix complexity and binding [36]. The threshold of hexanal in oil is equal to 320 or 75 μg/kg depending on whether it is determined orthonasally or retronasally, and an odour described as “tallowy” or “green leafy” [35]. The same authors have reported an odour threshold for pentanal in oil that is equal to 240 μg/kg (nasal) or 150 μg/kg (retronasal), described as “pungent” or “bitter almonds” [35]. At the same time, odour thresholds of pentanal and hexanal in infant formulas have exhibited odour thresholds as low as 12 and 4.5 μg/kg, respectively [37], which were substantially lower than the concentrations determined in this study (hexanal: 730 to 92,143 μg/kg, pentanal: 63 to 101,202 μg/kg). 

Hexanal started to increase sharply after 69 days of accelerated storage and increased further after 90 days (Figure 3h, Table A9 and Table A10). After 69 days, the maximum levels of the compound were observed for the control samples (24,737 μg/kg) and were significantly (*p* < 0.05) higher than all the supplemented with natural and synthetic antioxidants FFMPs, in which lower rates of hexanal formation were observed. The potential of the added antioxidants in FFMPs to control the rate of lipid oxidation could be ranked in the following decreasing order: 77 ppm of c: ca: ra (9675 μg/kg) > 200 ppm of c: ca: ra (7666 μg/kg) > 308 ppm of c: ca: ra (5541 μg/kg) > 200 ppm of BHA: BHT (3306 μg/kg), whereas 308 ppm of c: ca: ra did not differ significantly (*p* > 0.05) from the samples with 200 ppm of BHA: BHT. The highest hexanal concentration was observed for control samples (92,143 μg/kg) after 90 days of storage, followed by 77 ppm of c: ca: ra (84,077 μg/kg), 200 ppm of c: ca: ra (66,064 μg/kg), 308 ppm of c: ca: ra (39,213 μg/kg) and finally the lowest in samples with 200 ppm of BHA: BHT (21,672 μg/kg). However, again the high variability of the control samples after 90 days of accelerated storage led to a lack of significance (*p* > 0.05) after comparison with the supplemented with antioxidants samples. Even though, the same apparent trend was observed concerning the effectiveness and potency of all the different antioxidant combinations to control hexanal formation. In addition, in that case, 308 ppm of c: ca: ra substantially reduced its formation, but to a lesser extent than 200 ppm of BHA: BHT (*p* < 0.05). Olmedo et al. [38] also observed that hexanal was present at very high levels of 10,000 to 80,000 µg/kg in sunflower oil samples that were supplemented with laurel, oregano, and rosemary EOs as natural antioxidants [38]. Therefore, the high levels of hexanal in this study fall within the range reported by the former authors. As it could be further speculated, the high variability of pentanal and hexanal in the highly oxidized control samples could be potentially attributed to the presence of extremely high levels of these volatile compounds, which could have led to insufficient time for equilibration in the headspace, as well as saturation of the SPME fibre. In addition, in a number of cases (i.e., for hexanal after 5 days of accelerated storage), a higher standard deviation compared to the mean was observed (Table A5 of Appendix A). Other studies, as of Stewart et al. [39] have found high variability in the levels of volatile compounds in milk and milk powder samples. This can again be attributable to the inherent variability of food systems that in parallel to the high sensitivity of GC-MS analysis, can lead to these effects.

The remaining eight volatile indicators also increased in all FFMPs during accelerated storage, following the same trends as discussed for hexanal and pentanal (Figure 3a–j and Table A4, Table A5, Table A6, Table A7, Table A8, Table A9 and Table A10). Even if these aldehydes, ketones, ketenes and alcohol compounds were present in lower concentrations compared to pentanal and hexanal, their presence at substantial levels is potentially significant and many of these, particularly those with one or more double bonds have much lower odour thresholds than their saturated equivalents. For instance, as a result of the presence of carbon-to-carbon double bonds, even relatively low concentrations 2,4-decadienal in a product can result in its unacceptability [40], as the compounds has a low threshold of 2 μg/kg in skim milk and is related to a “fried fatty” and “painty odour” [41]. 2-heptanone has been established as a good indicator of “grassy flavor”, while 3-octen-2-one and octanal have been established as accurate indicators of “painty flavor” in whole milk powders [42]. The odour of heptanal is described as “oily” or “fishy” [35], as well as “woody” [37], whereas its threshold is 3200 μg/kg (nasal) or 50 μg/kg (retronasal) [35]. In respect to 1-pentanol, a low threshold (0.47 mg/kg) has also been found in oil [43]. Volatile compounds such as 3-octen-2-one and octanal that originate from C18:2 and C18:1 FAs, respectively, are not only formed due to the spontaneous decomposition of LOOH, but also due to the autoxidation throughout the production and storage of milk powders [34]. 

At this point, it is important to highlight that the HS-SPME-GC-MS method in this study has been designed to accurately quantify the determined volatiles in a range from 0 to 3000 μg/kg, which is normally sufficient for most matrices with present oxidized lipids. However, samples stored for 41, 69 and 90 days exceeded this range. This was especially evident for hexanal and pentanal after 69 and 90 days of accelerated storage. Hence, a higher concentration of internal standard was utilized to quantify these samples. Even so, there is a possibility of overloading of the SPME fibre, and, therefore, an underestimation of the levels of aldehydes after 69 and 90 days of accelerated storage. However, the limited capacity of SPME fibres is a known weakness of this technique [44].

## 4. Conclusions

The obtained results indicated the efficiency of all the different combinations of antioxidants to retard lipid oxidation after accelerated storage (40 °C, RH 23%) for 90 days and stabilise FFMPs, but with different degrees of efficacy when compared to control samples. The antioxidant potency of c: ca: ra (0.25: 0.38: 0.37) showed a concentration-related relationship (308 ppm > 200 ppm > 77 ppm), with the highest concentration being the most efficient at controlling the formation of TBARS and PVs. 308 ppm of c: ca: ra was also considerably effective at controlling oxidative volatile compounds’ developments, including the highly abundant hexanal and pentanal, as determined by HS-SPME-GC-MS analysis. Hence, 308 ppm were particularly effective in inhibiting all the evaluated oxidation products, even if it was, in general, less effective, and in some cases significantly (*p* < 0.05), than 200 ppm of BHA: BHT (1: 1). Utilising a standardised ratio of pure phenolic compounds in this study will enable not only the accurate reproduction of their effect in the future but also the elimination of any possibility of the exerted antioxidant effect to be attributable to additional compounds present. This constitutes a major issue that arises after the application of whole plant extracts in several studies. In summary, c: ca: ra may act as an alternative to synthetic antioxidants for the industrial manufacturing of FFMPs. The use of these potent compounds of rosemary may, therefore, offer several benefits to the nutritional value, preservation and safety of these dairy products. However, further studies utilising the specified c: ca: ra at higher concentrations could confirm the hypothesis that at a concentration above 308 ppm, these compounds could exert a similar or an even superior potency than this of 200 ppm of BHA: BHT. However, a prooxidant effect could also be observed since in high doses, antioxidants could adversely promote the oxidation process. The parallel organoleptic evaluation of the produced samples could determine any adverse effects of the high concentrations of these phenolics (i.e., bitter and astringent taste) and of potentially produced volatile aroma compounds (i.e., rancid aroma/warmed-over flavour) in the organoleptic characteristics of the final product. 

## Figures and Tables

**Table 1:** Particle size and statistical analysis output for the combined skim milk with sunflower oil (fat fraction) prior to spray drying, with or without the addition of antioxidant agents ^1, 2, 3, 4, 5^.

	D (10)	D (50)	D (90)	Span Index
Control	0.556 ± 0.01 ^a^	1.031 ± 0.03 ^a^	3.110 ± 0.09 ^a^	2.48 ^a^
200 ppm BHA: BHT	0.561 ± 0.01 ^a^	1.069 ± 0.06 ^a^	3.307 ± 0.43 ^a^	2.57 ^a^
77 ppm c: ca: ra	0.555 ± 0.04 ^a^	1.080 ± 0.02 ^a^	3.173 ± 0.11 ^a^	2.42 ^a^
200 ppm c: ca: ra	0.543 ± 0.03 ^a^	1.003 ± 0.02 ^a^	3.067 ± 0.05 ^a^	2.51 ^a^
308 ppm c: ca: ra	0.555 ± 0.00 ^a^	1.027 ± 0.05 ^a^	2.950 ± 0.08 ^a^	2.33 ^a^

^1^ Values are the mean of three replicates (*n* = 3) ±SD; ^2^ The data for D (10), D (50), and D (90) are expressed in μm; ^3^ c: ca: ra, carnosol: carnosic acid: rosmarinic acid; ^4^ D, Diameter; ^5^ As values in the same row for each different sampling day independently, share a letter (a), are not significantly different (*p* < 0.05).

**Table 2:** Changes of the L* coordinate (lightness), a* coordinate (redness/greenness) and b* coordinate (yellowness/blueness) of FFMPs, before and after accelerated storage (40 °C, RH 23%), with or without the addition of antioxidants ^1, 2, 3^.

Compound	Sample				
	Control	200 ppm BHA: BHT	77 ppm c: ca: ra	200 ppm c: ca: ra	308 ppm c: ca: ra
**Day 0**					
L*	96.81 ± 0.12 ^a^	97.34 ± 0.42 ^a^	97.2 ± 0.43 ^a^	97.08 ± 0.28 ^a^	97.22 ± 0.28^a^
a*	−2.84 ± 0.05 ^a^	−2.83 ± 0.07 ^a^	−2.94 ± 0.10 ^a^	−3.01 ± 0.04 ^a^	−3.14 ± 0.05 ^a^
b*	8.53 ± 0.15 ^a^	8.22 ± 0.12 ^a^	8.64 ± 0.38 ^a^	9.14 ± 0.52 ^a^	9.02 ± 0.39 ^a^
**Day 5**					
L*	97.03 ± 0.20 ^a^	97.54 ± 0.17 ^a^	97.11 ± 0.10 ^a^	96.94 ± 0.54 ^a^	97.22 ± 0.18 ^a^
a*	−2.80 ± 0.11 ^a^	−2.90 ± 0.07 ^a^	−3.16 ± 0.03 ^b^	−3.05 ± 0.07 ^b^	−3.14 ± 0.15 ^b^
b*	8.16 ± 0.20 ^a^	8.39 ± 0.20 ^a^	9.15 ± 0.10 ^b^	8.94 ± 0.17 ^b^	9.24 ± 0.00 ^b^
**Day 13**					
L*	97.59 ± 0.37 ^a^	97.37 ± 0.13 ^a^	97.16 ± 0.05 ^a^	97.35 ± 0.25 ^a^	97.33 ± 0.21 ^a^
a*	−2.77 ± 0.06 ^a^	−2.79 ± 0.07 ^a^	−3.01 ± 0.03 ^ab^	−2.84 ± 0.13 ^ab^	−3.14 ± 0.13 ^b^
b*	8.34 ± 0.30 ^a^	8.47 ± 0.20 ^a^	8.80 ± 0.27 ^a^	8.74 ± 0.33 ^a^	9.51 ± 0.12 ^b^
**Day 27**					
L*	97.04 ± 0.43 ^a^	97.25 ± 0.42 ^a^	96.59 ± 0.15 ^a^	97.18 ± 0.18 ^a^	96.77 ± 0.16 ^a^
a*	−2.64 ± 0.09 ^a^	−2.66 ± 0.07 ^a^	−2.89 ± 0.04 ^bc^	−2.78 ± 0.08 ^ab^	−2.94 ± 0.05 ^c^
b*	7.91 ± 0.37 ^a^	8.36 ± 0.27 ^ab^	8.94 ± 0.02 ^bc^	8.60 ± 0.23 ^bc^	9.23 ± 0.10 ^c^
**Day 41**					
L*	97.38 ± 0.30 ^ab^	97.53 ± 0.27 ^a^	97.25 ± 0.07 ^ab^	97.33 ± 0.36 ^ab^	96.73 ± 0.14 ^b^
a*	−2.54 ± 0.06 ^a^	−2.59 ± 0.18 ^ab^	−2.94 ± 0.06 ^b^	−2.76 ± 0.08 ^a^	−2.90 ± 0.05 ^b^
b*	7.99 ± 0.19 ^a^	8.25 ± 0.37 ^ab^	9.02 ± 0.15 ^c^	8.77 ± 0.09 ^bc^	9.09 ± 0.13 ^c^
**Day 69**					
L*	97.21 ± 0.16 ^a^	97.47 ± 0.33 ^a^	97.24 ± 0.25 ^a^	96.70 ± 0.23 ^a^	96.83 ± 0.25 ^a^
a*	−2.53 ± 0.09 ^a^	−2.70 ± 0.00 ^ab^	−2.81 ± 0.04 ^bc^	−2.79 ± 0.04 ^abc^	−2.83 ± 0.04 ^c^
b*	8.59 ± 0.11 ^a^	8.56 ± 0.08 ^a^	9.17 ± 0.09 ^a^	8.91 ± 0.16 ^a^	9.23 ± 0.10 ^a^
**Day 90**					
L*	97.00 ± 0.26 ^ab^	97.39 ± 0.10 ^b^	96.76 ± 0.06 ^ab^	96.58 ± 0.31 ^a^	96.47 ± 0.39 ^a^
a*	−2.72 ± 0.03 ^a^	−2.78 ± 0.11 ^a^	−2.82 ± 0.09 ^a^	−2.76 ± 0.11 ^a^	−2.87 ± 0.06 ^a^
b*	10.16 ± 0.46 ^a^	9.34 ± 0.28 ^b^	10.43 ± 0.31 ^a^	9.70 ± 0.16 ^ab^	9.75 ± 0.21 ^ab^

^1^ Values are the mean of three replicates (n = 3) ± SD; ^2^ c: ca: ra, carnosol: carnosic acid: rosmarinic acid; ^3^ Values in the same row for each different sampling day independently, which do not share a letter (^a–c^), are significantly different (*p* < 0.05).

## Data Availability

Data is contained within the article.

## Appendix A

**Table A1:** PVs of the FFMPs after accelerated storage (40 °C, RH 23%) for 69 and 90 days, with or without the addition of antioxidants ^1, 2, 3, 4^.

	PVs	
	Days	
Sample	69	90
Control	222.22 ± 52.35 ^a^	285.40 ± 12.10 ^a^
200 ppm BHA: BHT	88.00 ± 7.42 ^b^	158.78 ± 8.01 ^b^
77 ppm c: ca: ra	161.33 ± 12.86 ^ac^	272.44 ± 11.50 ^a^
200 ppm c: ca: ra	104.89 ± 2.04 ^ac^	264.00 ± 10.41 ^a^
308 ppm c: ca: ra	92.44 ± 8.47 ^bc^	219.90 ± 24.04 ^c^

^1^ Values are the mean of three replicates (*n* = 3) ± SD.; ^2^ c: ca: ra, carnosol: carnosic acid: rosmarinic acid; ^3^ Values in the same column, which do not share a letter (^a–c^), are significantly different (*p* < 0.05); ^4^ Results are expressed as meq/kg of fat.

**Table A2:** TBARS of the FFMPs before and after accelerated storage (40 °C, RH 23%), with or without the addition of antioxidants ^1, 2, 3, 4^.

	TBARS						
	Days						
Sample	0	5	13	27	41	69	90
Control	0.00	2.72 ± 0.47 ^a^	4.39 ± 0.18 ^a^	10.73 ± 0.92 ^a^	9.71 ± 1.16 ^a^	72.14 ± 23.32 ^a^	156.55 ± 9.92 ^a^
200 ppm BHA: BHT	0.00	3.10 ± 0.62 ^a^	3.30 ± 0.82 ^ab^	9.00 ± 0.41 ^b^	7.20 ± 0.06 ^b^	13.07 ± 1.13 ^b^	42.42 ± 1.38 ^b^
77 ppm c: ca: ra	0.00	3.17 ± 0.41 ^a^	2.72 ± 0.24 ^b^	8.93 ± 0.33 ^b^	7.71 ± 0.31 ^b^	21.76 ± 3.03 ^ab^	134.94 ± 4.68 ^c^
200 ppm c: ca: ra	0.00	2.83 ± 0.48 ^a^	2.72 ± 0.31 ^b^	8.12 ± 0.33 ^b^	6.56 ± 0.62 ^bc^	13.95 ± 1.59 ^ab^	98.81 ± 7.18 ^d^
308 ppm c: ca: ra	0.00	2.62 ± 0.06 ^a^	3.23 ± 0.68 ^ab^	8.25 ± 0.10 ^b^	5.47 ± 0.06 ^c^	12.16 ± 0.82 ^b^	72.28 ± 3.30 ^e^

^1^ Values are the mean of three replicates (*n* = 3) ± SD; ^2^ c: ca: ra, carnosol: carnosic acid: rosmarinic acid; ^3^ Values in the same column, which do not share a letter (^a–e^), are significantly different (*p* < 0.05); ^4^ Results are expressed as μΜ MDA/100 g FFMP.

**Table A3:** Correlation *r_s_* between TBARS and individual volatile compounds (1-pentanol, 2-heptanone, 2-pentanone, 3-octane-2-one, 2,4-decadienal, hexanal, heptanal, octanal, pentanal, 2-nonenal) ^3^.

Volatile Compounds after HS-SPME-GC-MS analysis ^2^										
	1-Pentanol	2-Heptanone	2-Pentanone	3-Octane-2-One	2,4-Decadienal	Hexanal	Heptanal	Octanal	Pentanal	2-Nonenal
TBARS ^1^	0.796	0.909	0.754	0.915	0.796	0.728	0.800	0.650	0.671	0.796

^1^ Results are expressed as μΜ MDA/100 g FFMP; ^2^ Results are expressed as μg/kg of FFMP; ^3^ *p* < 0.01.

**Table A4:** Levels of volatile compounds (μg/kg of FFMP) as measured with HS-SPME-GC-MS analysis in FFMPs with or without antioxidants, before accelerated storage (40 °C, RH 23%) ^1, 2, 3, 4^.

Day 0					
Compound	Sample				
	Control	200 ppm BHA: BHT	77 ppm c: ca: ra	200 ppm c: ca: ra	308 ppm c: ca: ra
1-pentanol	0.0	0.0	0.0	0.0	0.0
2-heptanone	27.4 ± 0.3 ^a^	27.3 ± 0.2 ^a^	27.6 ± 0.5 ^a^	27.4 ± 0.4 ^a^	27.2 ± 0.1 ^a^
2-pentanone	0.0	0.0	0.0	0.0	0.0
3-octen-2-one	39.4 ± 1.7 ^a^	36.9 ± 1.6 ^ab^	36.1 ± 0.2 ^ab^	35.0 ± 1.3 ^b^	35.2 ± 1.2 ^b^
2,4-decadienal	0.0	0.0	0.0	0.0	0.0
hexanal	2419.4 ± 505.5 ^a^	1021.4 ± 203.8 ^b^	2503.7 ± 415.2 ^a^	2135.6 ± 201.7 ^a^	1903.0 ± 535.6 ^ab^
heptanal	59.8 ± 4.9 ^a^	35.4 ± 3.8 ^a^	36.1 ± 7.1 ^a^	41.6 ± 6.3 ^a^	45.2 ± 13.5 ^a^
octanal	79.0 ± 8.2 ^a^	50.4 ± 5.2 ^ab^	42.4 ± 7.7 ^b^	57.1 ± 24.2 ^ab^	46.2 ± 3.1 ^ab^
pentanal	337.9 ± 30.9 ^a^	134.8 ± 21.0 ^b^	169.4 ± 23.6 ^b^	169.1 ± 25.8 ^b^	163.4 ± 32.7 ^b^
2-nonenal, (E)	0.0	0.0	0.0	0.0	0.0

^1^ Values are the mean of three replicates (*n* = 3) ± SD; ^2^ c: ca: ra, carnosol: carnosic acid: rosmarinic acid; ^3^ Values in the same row, which do not share a letter (^a,b^), are significantly different (*p* < 0.05); ^4^ Results are expressed as μg/kg of FFMP.

**Table A5:** Levels of volatile compounds (μg/kg of FFMP) as measured with HS-SPME-GC-MS analysis in FFMPs with or without antioxidants, after accelerated storage (40 °C, RH 23%) for 5 days ^1, 2, 3, 4^.

Day 5					
Compound	Sample				
	Control	200 ppm BHA: BHT	77 ppm c: ca: ra	200 ppm c: ca: ra	308 ppm c: ca: ra
1-pentanol	0.0	0.0	0.0	0.0	0.0
2-heptanone	27.7 ± 1.0 ^a^	27.7 ± 0.4 ^a^	27.3 ± 0.6 ^a^	27.3 ± 0.5 ^a^	27.6 ± 0.5 ^a^
2-pentanone	0.0	0.0	0.0	0.0	0.0
3-octen-2-one	53.2 ± 1.0 ^a^	48.2 ± 5.7 ^ab^	43.1 ± 1.7 ^b^	39.0 ± 2.5 ^b^	38.9 ± 1.1 ^b^
2,4-decadienal	0.0	0.0	0.0	0.0	0.0
hexanal	2347.1 ± 540.1 ^a^	729.7± 51.4 ^a^	1926.0 ± 788.5 ^a^	1402.3 ± 764.3 ^a^	1858.9 ± 1972.8 ^a^
heptanal	41.6 ± 4.0 ^a^	36.8 ± 1.3 ^a^	48.1 ± 21.9 ^a^	45.2 ± 17.9 ^a^	61.5 ± 48.5 ^a^
octanal	64.3 ± 10.2 ^a^	58.3 ± 6.4 ^a^	242.5 ± 327.9 ^a^	46.4 ± 2.3 ^a^	47.3 ± 0.5 ^a^
pentanal	144.4 ± 19.6 ^a^	102.7 ± 23.9 ^ab^	62.7 ± 39.9 ^b^	98.7 ± 9.7 ^ab^	92.0 ± 13.4 ^ab^
2-nonenal, (E)	0.0	0.0	0.0	0.0	0.0

^1^ Values are the mean of three replicates (*n* = 3) ± SD; ^2^ c: ca: ra, carnosol: carnosic acid: rosmarinic acid; ^3^ Values in the same row, which do not share a letter (^a,b^), are significantly different (*p* < 0.05); ^4^ Results are expressed as μg/kg of FFMP.

**Table A6:** Levels of volatile compounds (μg/kg of FFMP) as measured with HS-SPME-GC-MS analysis in FFMPs with or without antioxidants, after accelerated storage (40°C, RH 23%) for 13 days ^1, 2, 3, 4^.

Day 13					
Compound	Sample				
	Control	200 ppm BHA: BHT	77 ppm c: ca: ra	200 ppm c: ca: ra	308 ppm c: ca: ra
1-pentanol	0.0	0.0	0.0	0.0	0.0
2-heptanone	29.0 ± 0.4 ^a^	28.2 ± 1.4 ^a^	26.8 ± 0.2 ^a^	27.1 ± 0.7 ^a^	27.9 ± 1.2 ^a^
2-pentanone	0.0	0.0	0.0	0.0	0.0
3-octen-2-one	74.7 ± 5.8 ^a^	79.5 ± 3.9 ^a^	88.8 ± 7.2 ^a^	57.2 ± 4.3 ^b^	80.7 ± 8.3 ^a^
2,4-decadienal	0.0	0.0	0.0	0	0.0
hexanal	1361.8 ± 28.6 ^ab^	1530.0 ± 195.2 ^b^	1397.4 ± 352.7 ^ab^	879.0 ± 87.2 ^a^	1014.8 ± 213 ^ab^
heptanal	43.6 ± 1.9 ^ab^	40.3 ± 0.9 ^b^	49.6 ± 2.5 ^a^	37.5 ± 1.2 ^b^	40.5 ± 3.7 ^b^
octanal	68.4 ± 1.2 ^a^	56.6 ± 1.9 ^ab^	62.4 ± 1.4 ^ab^	48.3 ± 1.2 ^b^	61.4 ± 11.4 ^ab^
pentanal	155.5 ± 2.6 ^ab^	131.9 ± 0.7 ^ab^	216.0 ± 46.1 ^b^	119.4 ± 2.0 ^a^	109.0 ± 33.0 ^a^
2-nonenal, (E)	0.0	0.0	0.0	0.0	0.0

^1^ Values are the mean of three replicates (*n* = 3) ± SD; ^2^ c: ca: ra, carnosol: carnosic acid: rosmarinic acid; ^3^ Values in the same row, which do not share a letter (^a,b^), are significantly different (*p* < 0.05); ^4^ Results are expressed as μg/kg of FFMP.

**Table A7:** Levels of volatile compounds (μg/kg of FFMP) as measured with HS-SPME-GC-MS analysis in FFMPs with or without antioxidants, after accelerated storage (40°C, RH 23%) for 27 days ^1, 2, 3, 4^.

Day 27					
Compound	Sample				
	Control	200 ppm BHA: BHT	77 ppm c: ca: ra	200 ppm c: ca: ra	308 ppm c: ca: ra
1-pentanol	0.0	0.0	0.0	0.0	0.0
2-heptanone	33.6 ± 0.1 ^a^	31.8± 0.4 ^b^	31.8 ± 1.2 ^abc^	31.7 ± 0.3 ^b^	30.3 ± 0.2 ^c^
2-pentanone	0.0	0.0	0.0	0.0	0.0
3-octen-2-one	245.4 ± 9.9 ^a^	198.4 ± 12.8 ^b^	192.2 ± 12.8 ^b^	193.4 ± 13.2 ^b^	120.4 ± 9.5 ^c^
2,4-decadienal	0.0	0.0	0.0	0.0	0.0
hexanal	3285.8 ± 373.4 ^a^	1504.3 ± 166.0 ^bc^	1862.0 ± 49.3 ^c^	1848.0 ± 126.0 ^c^	1249.3 ± 104.7 ^b^
heptanal	56.7 ± 1.0 ^a^	43.4 ± 1.3 ^bc^	41.0 ± 0.8 ^c^	46.3 ± 2.5 ^b^	41.3 ± 1.2 ^c^
octanal	52.3 ± 2.5 ^a^	51.3 ± 13.3 ^ab^	40.4 ± 2.1 ^ab^	49.4 ± 2.2 ^a^	35.2 ± 1.9 ^b^
pentanal	223.1 ± 27.2 ^ac^	122.5 ± 7.1 ^a^	122.4 ± 3.1 ^ab^	151.7 ± 4.6 ^cb^	101.2 ± 7.9 ^b^
2-nonenal, (E)	0.0	0.0	0.0	0.0	0.0

^1^ Values are the mean of three replicates (*n* = 3) ± SD; ^2^ c: ca: ra, carnosol: carnosic acid: rosmarinic acid; ^3^ Values in the same row, which do not share a letter (^a–c^), are significantly different (*p* < 0.05); ^4^ Results are expressed as μg/kg of FFMP.

**Table A8:** Levels of volatile compounds (μg/kg of FFMP) as measured with HS-SPME-GC-MS analysis in FFMPs with or without antioxidants, after accelerated storage (40 °C, RH 23%) for 41 days ^1, 2, 3, 4^.

Day 41					
Compound	Sample				
	Control	200 ppm BHA: BHT	77 ppm c: ca: ra	200 ppm c: ca: ra	308 ppm c: ca: ra
1-pentanol	0.0	0.0	0.0	0.0	0.0
2-heptanone	49.1 ± 1.1 ^a^	35.1 ± 0.9 ^c^	44.5 ± 3.2 ^ab^	41.7 ± 3.1 ^b^	34.2 ± 0.7 ^c^
2-pentanone	0.0	0.0	0.0	0.0	0.0
3-octen-2-one	676.9 ± 67.2 ^a^	387.3 ± 7.2 ^b^	678.7 ± 60.9 ^a^	624.6 ± 41.7 ^a^	273.4 ± 11.9 ^b^
2,4-decadienal	0.0	0.0	0.0	0.0	0.0
hexanal	6410.1 ± 511.9 ^a^	2418.8 ± 138.4 ^b^	5044.3 ± 474.3 ^c^	4850.7 ± 818.9 ^c^	2389.5 ± 286.1 ^b^
heptanal	75.1 ± 3.4 ^a^	46.4 ± 2.8 ^b^	66.5 ± 5.2 ^a^	64.5 ± 6.6 ^a^	47.1 ± 1.4 ^b^
octanal	82.3 ± 11.4 ^abc^	60.7 ± 10.4 ^abc^	86.0 ± 19.5 ^abc^	81.2 ± 1.7 ^b^	47.8 ± 1.1 ^c^
pentanal	481.9 ± 5.1 ^a^	159.4 ± 21.5 ^b^	317.4 ± 29.0 ^c^	327.8 ± 22.6 ^c^	142.7 ± 6.5 ^b^
2-nonenal, (E)	0.0	0.0	0.0	0.0	0.0

^1^ Values are the mean of three replicates (*n* = 3) ± SD; ^2^ c: ca: ra, carnosol: carnosic acid: rosmarinic acid; ^3^ Values in the same row, which do not share a letter (^a–c^), are significantly different (*p* < 0.05); ^4^ Results are expressed as μg/kg of FFMP.

**Table A9:** Levels of volatile compounds (μg/kg of FFMP) as measured with HS-SPME-GC-MS analysis in FFMPs with or without antioxidants, after accelerated storage (40 °C, RH 23%) for 69 days ^1, 2, 3, 4^.

Day 69					
Compound	Sample				
	Control	200 ppm BHA: BHT	77 ppm c: ca: ra	200 ppm c: ca: ra	308 ppm c: ca: ra
1-pentanol	2325.9 ± 175.3 ^a^	140.7 ± 25.4 ^b^	893.9 ± 161.0 ^c^	592.3 ± 43.6 ^cd^	360.1 ± 97.1 ^bd^
2-heptanone	534.3 ± 113.1 ^abc^	70.6 ± 16.3 ^a^	284.2 ± 47.6 ^cd^	189.9 ± 44.1 ^abd^	149.5 ± 21.4 ^bd^
2-pentanone	28.1 ± 7.0 ^a^	2.7 ± 2.8 ^b^	9.0 ± 10.4 ^ab^	11.3 ± 9.9 ^ab^	11.4 ± 7.9 ^ab^
3-octen-2-one	1798.1 ± 970.6 ^a^	244.4 ± 132.4 ^a^	715.5 ± 235 ^a^	381.3 ± 78.2 ^a^	356.3 ± 81.1 ^a^
2,4-decadienal	88.1 ± 65.6 ^a^	3.4 ± 4.0 ^a^	9.5 ± 7.6 ^a^	2.8 ± 0.8 ^a^	5.1 ± 3.9 ^a^
hexanal	24,737.4 ± 2134.8 ^a^	3305.5 ± 568.8 ^b^	9674.6 ± 2258.0 ^c^	7665.8 ± 692.4 ^c^	5540.7 ± 1304.8 ^bc^
heptanal	3044.9 ± 458.6 ^a^	359.0 ± 74.0 ^b^	1068.1 ± 269.2 ^c^	848.2 ± 72.6 ^bc^	606.5 ± 134.0 ^bc^
octanal	2852.8 ± 308.0 ^a^	165.7 ± 63.0 ^b^	651.6 ± 111.8 ^c^	407.9 ± 62.9 ^c^	342.6 ± 43.0 ^bc^
pentanal	14,163.7 ± 1911.8 ^a^	1331.4 ± 94.0 ^b^	4713.3 ± 887.5 ^bc^	3619.2 ± 279.0 ^cd^	2649.1 ± 523.3 ^bd^
2-nonenal, (E)	132.3 ± 58.9 ^a^	3.3 ± 2.5 ^a^	18.4 ± 6.6 ^a^	7.1 ± 4.4 ^a^	6.9 ± 1.5 ^a^

^1^ Values are the mean of three replicates (*n* = 3) ± SD; ^2^ c: ca: ra, carnosol: carnosic acid: rosmarinic acid; ^3^ Values in the same row, which do not share a letter (^a–d^), are significantly different (*p* < 0.05); ^4^ Results are expressed as μg/kg of FFMP.

**Table A10:** Levels of volatile compounds (μg/kg of FFMP) as measured with SPME-GC-MS analysis in FFMPs with or without antioxidants, after accelerated storage (40 °C, RH 23%) for 90 days ^1, 2, 3, 4^.

Day 90					
Compound	Sample				
	Control	200 ppm BHA: BHT	77 ppm c: ca: ra	200 ppm c: ca: ra	308 ppm c: ca: ra
1-pentanol	16,285.1 ± 1132.2 ^a^	4796.2 ± 524.5 ^c^	17,399.9 ± 1670.0 ^a^	13,028.3 ± 1013.5 ^b^	7491.0 ± 314.2 ^c^
2-heptanone	7031.9 ± 114.0 ^a^	1223.1 ± 144.1 ^b^	6229.0 ± 133.1 ^c^	4334.0 ± 173.7 ^d^	2295.7 ± 126.6 ^e^
2-pentanone	615.4 ± 56.8 ^a^	118.7 ±17.9 ^c^	588.2 ± 83.3 ^a^	385.1 ± 49.0 ^b^	208.5 ± 9.6 ^c^
3-octen-2-one	6830.0 ± 334.9 ^a^	2605.7 ± 179.1 ^a^	8345.1 ± 379.1 ^b^	6616.7 ± 295.3 ^c^	4245.7 ± 416.9 ^d^
2,4-decadienal	5776.7 ± 1446.8 ^abc^	130.4 ± 43.2 ^a^	4154.3 ± 554.6 ^b^	3375.9 ± 626.0 ^b^	511.3 ± 80.0 ^c^
hexanal	92,143.4 ± 22,255 ^abc^	21,671.8 ± 1673.4 ^a^	84,076.5 ± 8359.1 ^b^	66,064.3 ± 10,161 ^bc^	39,213.7 ± 1997.5 ^c^
heptanal	14,984.6 ± 3773.8 ^abc^	4384.8 ± 569.4 ^a^	12,071.9 ± 1603.4 ^b^	9638.1 ± 1560.8 ^abc^	5614.6 ± 293.9 ^c^
octanal	17,116.9 ± 4028.1 ^abc^	4384.8 ± 569.4 ^b^	12,071.9 ± 1603.4 ^c^	9638.1 ± 1561 ^bc^	5614.6 ± 293.9 ^bc^
pentanal	101,202.4 ± 22,253.8 ^abc^	19,116.7 ± 1442.0 ^a^	84,637.9 ± 6732.3 ^b^	62,672.6 ± 11,168.8 ^abc^	36,137.7 ± 1988.5 ^c^
2-nonenal, (E)	2425.9 ± 462.4 ^a^	126.1 ± 11.8 ^b^	1919.8 ± 288.1 ^a^	1371.0 ± 181.7 ^a^	339.2 ± 54.0 ^b^

^1^ Values are the mean of three replicates (*n* = 3) ± SD; ^2^ c: ca: ra, carnosol: carnosic acid: rosmarinic acid; ^3^ Values in the same row, which do not share a letter (^a–d^), are significantly different (*p* < 0.05); ^4^ Results are expressed as μg/kg of FFMP.
